# Allogeneic Hematopoietic Stem Cell Transplantation in a Rare Case of Tonsillar Mast Cell Sarcoma

**DOI:** 10.3389/fonc.2020.00219

**Published:** 2020-02-28

**Authors:** Anne Sophie Kubasch, Georg-Nikolaus Franke, Ali Aldaoud, Konstantin Weibl, Madlen Jentzsch, Osama Sabri, Hans-Peter Horny, Falko Fend, Gerhard Behre, Uwe Platzbecker, Vladan Vucinic

**Affiliations:** ^1^Medical Clinic and Policlinic 1, Hematology and Cellular Therapy, University Hospital Leipzig, Leipzig, Germany; ^2^Outpatient-Centre for Hematology and Oncology, Leipzig, Germany; ^3^Department of Nuclear Medicine, University Hospital Leipzig, Leipzig, Germany; ^4^Institute of Pathology, Ludwig-Maximilians University Munich, Munich, Germany; ^5^Institute of Pathology and Neuropathology, University of Tuebingen and Comprehensive Cancer Center, University Hospital Tuebingen, Tuebingen, Germany

**Keywords:** mast cell sarcoma, allogeneic stem cell transplantation, mast cell neoplasm, rare disease (RD), poor prognosis

## Abstract

Mast cell sarcoma comprises a rare aggressive mast cell neoplasia with histological, clinical, and genetic features distinct from other mast cell neoplasm. Until now, prognosis is still poor due to high rates of progression to mast cell leukemia and failure of conventional chemotherapies. Our here presented first report about successful allogeneic hematopoietic stem cell transplantation leading to remission in a case of tonsillar MCS represents a promising potential curative treatment option for this rare and often fatal disease.

## Key-Points

- Mast cell sarcoma (MCS) is a rare, aggressive mast cell neoplasia with a very poor prognosis and a median survival of <18 months.
- Allo-HSCT represents a promising potential curative treatment option for this rare and often fatal disease.

## Introduction

Mast cell sarcoma (MCS) is a rare, aggressive mast cell neoplasia characterized by cytological malignant mast cells comprising a solid tumor with destructive capability and metastatic potential (1). Due to high rates of progression to mast cell leukemia and failure of conventional chemotherapies, prognosis of MCS patients is very poor with a median survival of <18 months (1, 2). Thus, the curative potential of new targeted treatment approaches and allogeneic hematopoietic stem cell transplantation is currently discussed in literature. We here report the first case of a female MCS patient achieving remission and disease-control after matched unrelated donor peripheral blood hematopoietic stem cell transplantation (MUD-PBSCT).

## Case Presentation

**Figure 1:**
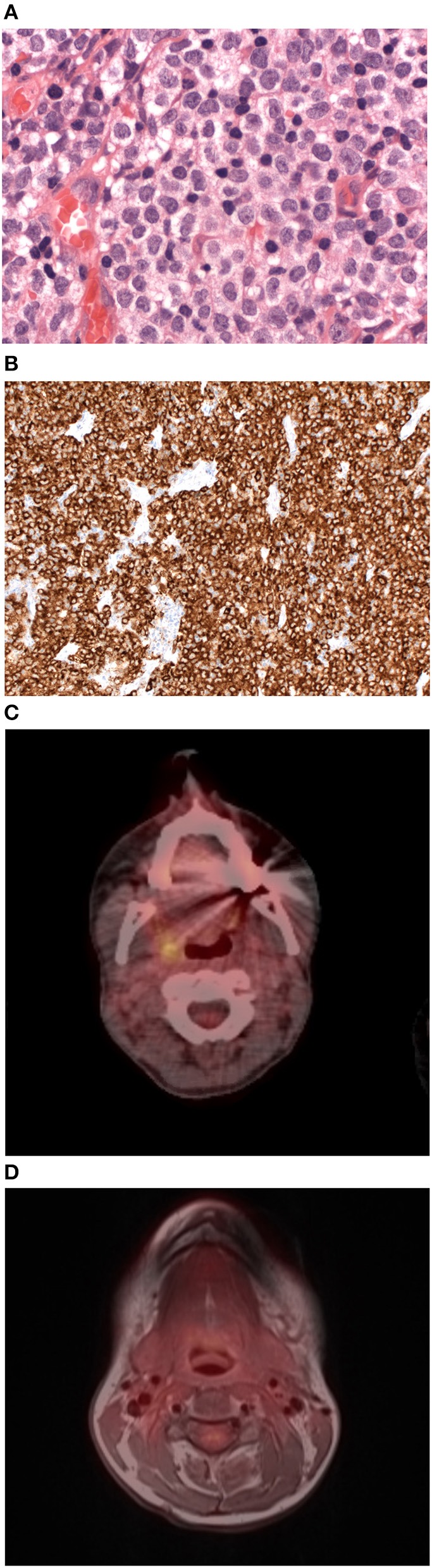
Histological and radiological features of tonsillar mast cell sarcoma. **(A)** Mast cell sarcoma cell staining with haematoxylin and eosin (HE) showing medium to large pleomorphic mast cells with large irregular nuclei (x400). **(B)** Mast cell sarcoma immunohistochemical staining for tryptase antigen (x400). **(C)** Axial/Coronar FDG-PET/CT scan of mast cell sarcoma performed after radiation therapy showing an increased FDG uptake in the remaining primary lesion of left and right tonsils (SUVmax = 5.0). **(D)** Axial/Coronar FDG-PET/MRI scan in mast cell sarcoma performed on day +147 after allogenic hematopoietic stem cell transplantation with no radiotracer uptake in the primary lesion in line with remission of MCS.

The 34 years old patient initially presented with swelling of left cervical lymph nodes and left palatine tonsil without significant clinical symptoms like fever, flush, malaise, or tachycardia. Cervical MRI scan showed high suspicion of malignancy, thus left cervical lymphadenectomy and targeted tonsillar biopsy was implemented. First histopathologic results revealed the suspicion of lymphoma. To confirm the diagnosis, a complete left tonsillectomy was intended, but due to bleeding complication during surgery, a local residual tumor remained (*R*^2^ tonsillar resection). Unexpectedly, local as well as reference histopathology results of left palatine tonsil confirmed a high proliferative mast cell neoplasia in line with the diagnosis of mast cell sarcoma (MCS) (Figure 1A). Immunohistochemical staining revealed malignant mast cell expression of tryptase antigen (Figure 1B) and CD117 (KIT), CD56 as well as partially aberrant CD25 and CD30. Moreover, the proliferative activity (MIB-1 index) was high with >50%. Laboratory results including serum tryptase levels were completely normal, the bone marrow (BM) smears showed normal blast count without significant mast cell infiltration. The BM karyotype was 46 XX, and the *KIT* D816V mutation was not detected in the BM. A whole-body PET/CT scan after partial left tonsillectomy was performed 1 h after the injection of standard activity (243,3 MBq) ^18^F–FDG. PET scan showed an increased FDG uptake in the remaining primary lesion of left and right tonsils (SUVmax = 5.0) as well as cervical retromandibular and dorsomedial radiotracer uptake (Figure 1C). One month after diagnosis of MCS, 7 MV Photon radiation therapy with a simultaneous integrated boost including Waldeyer's tonsillar ring and bilaterally cervical lymph drainage region was initiated (single doses of 2.5 Gy five times a week until a total dose of 50.0 Gy). Two months after finalization of radiotherapy, PET-CT scan still showed an increased FDG uptake of the right tonsil (SUVmax = 5.8, Figure 1), right cervical retromandibular (SUVmax = 3.4) and diffuse ^18^F-FDG bone marrow uptake in line with persisting activity of MCS after radiation therapy. Repeated BM biopsy showed again no significant mast cell infiltration in line with no progression to mast cell leukemia.

Due to her constant good general condition, the patient underwent human leukocyte antigen (HLA)- matched unrelated donor peripheral blood hematopoietic stem cell transplantation 5 months after initial diagnosis of MCS. Conditioning regimen consisted of fludarabine 30 mg/m^2^ on days −8 to −3 (total dose 54,4 mg/d), busulfane 0.8 mg/kg × 4/d on days −7 to −5 (total dose 538,2 mg) and ATG Neovii 20 mg/kg days −3 to −1 (total dose 1,340 mg/d). GvHD prophylaxis consisted of cyclosporine starting day −1 (5 mg/kg/d) and methotrexate on days +1, +3, and +6 (10 ng/m^2^; total dose 18, 1 mg/d). The patient achieved primary neutrophil engraftment on day 16. Quantitative chimerism analysis was performed on day 28 post-allogeneic hematopoietic stem cell transplantation by short-tandem repeat-based PCR techniques and confirmed a complete (100%) donor chimerism in the BM.

On day +34, whole body PET/MRI scan showed symmetrical physiological enhancement of ^18^F-FDG at Waldeyer's tonsillar ring without increased FDG uptake at left and right tonsillar region or cervical lymph nodes, in line with no evidence of local recurrence of MCS after allogeneic hematopoietic stem cell transplantation. In order to reduce the relapse risk, a strategy of early tapering of cyclosporine was followed and on day +92 we were able to discontinue cyclosporine therapy without any signs of significant acute graft vs. host disease (aGvHD). Three weeks after the end of immunosuppressive treatment, an episode of nausea, maculopapular rash, diarrhea, and elevated liver enzymes occurred. Histopathological analysis after gastroscopy and colonoscopy revealed typical aGvHD lesions, in line with confirmation of grade 3 [MAGIC criteria (3)] aGvHD involving the upper- and lower- gastrointestinal tract, liver and skin. Thus, glucocorticoid treatment (2 mg/kg body weight) was given for 1 week, the dose was tapered over the following week and cyclosporine was restarted. On day +147 whole body PET/MRI scan was repeated and showed again physiological ^18^F-FDG uptake at left and right tonsillar region, cervical lymph nodes, and physiological radiotracer uptake at the remaining investigated areas, confirming the complete remission of MCS (Figure 1D). Eleven months (day +337) after allogeneic hematopoietic stem cell transplantation, the patient is alive without any evidence for relapse of MCS.

## Discussion

In summary, MCS comprises a rare aggressive mast cell neoplasm with histological, clinical, and genetic features distinct from other mast cell neoplasm. Thus, MCS still represents a diagnostic challenge and was first describes 1986 by Horny et al. (4). To our knowledge, our case is the first report about a patient with tonsillar MCS achieving remission after allogeneic hematopoietic stem cell transplantation. The 2016 WHO classification defines MCS as a variant of mastocytosis, which presents as a unifocal mast cell tumor with destructive growth and high-grade cytology without multifocal infiltration of mast cells in bone marrow, skin, or other organs (5). The disease is relatively common in animals but extremely rare in humans with only a few cases reported in literature (*n* ~ 25). Review of available literature about MCS cases indicates, that age at presentation ranges from 1 to 77 years with a median age at diagnosis of 41 years (1) and affected regions range from lips and subglottis, gastrointestinal tract, uterus, bone, or skin. Thus, to our knowledge this is the first reported case of tonsillar MCS. Because of the rarity of MCS, targeted diagnostic features and efficient therapeutic regimens are not yet established. As described in our case, *KIT* D816V mutation is not common and reported in only 20% of MCS cases. Clinical presentation of MCS is variable and diagnosis is complicated due to multiple differential diagnoses. Thus, most of the MCS cases described in literature have been initially misdiagnosed as lymphomas, mastocytosis, langerhans cell histiocytosis, or acute myeloid leukemia. Mast cell neoplasms with *KIT* D816V mutation have been described as resistant to tyrosine kinase inhibitors like imatinib (6). In *KIT* D816V WT patients like in our case, imatinib may serve as possible effective treatment option (7) in case of MCS relapse. Other tyrosine kinase inhibitors like dasatinib or midostaurin, Interferon-α as well as 2-Chlorodeoxyadenosine (2-CdA) demonstrated no or only short-term effects in patients with MCS (1, 8). In line with our report, radiation therapy showed very limited evidence for efficacy in previous MCS cases (2). Moreover, the use of common chemotherapy regimens appropriate for lymphoid or myeloid neoplasm did also not demonstrate long-term responses, in only one MCS case a short transient response after chemotherapy was reported (1). After excision surgery, remission of limited duration in localized disease stage has been described (1, 8).

Thus until today, due to very limited treatment options, a very short median survival and high rates of progression to mast cell leukemia, the prognosis of MCS patients is very poor (1, 2). Despite the short follow-up, our here presented first report about successful allogeneic hematopoietic stem cell transplantation in a case of tonsillar MCS represents a promising potential curative treatment option for this rare and often fatal disease.

## Ethics Statement

Written informed consent was obtained from the individual(s) for the publication of any potentially identifiable images or data included in this article.

## Author Contributions

AK and UP wrote the manuscript. AK, VV, AA, KW, MJ, OS, H-PH, FF, GB, G-NF, and UP were responsible for patient care and provided clinical data. All authors reviewed, edited, and approved the final manuscript.

### Conflict of Interest

The authors declare that the research was conducted in the absence of any commercial or financial relationships that could be construed as a potential conflict of interest.
